# Participation in organized leisure-time activities and risk behaviors in Czech adolescents

**DOI:** 10.1007/s00038-016-0930-9

**Published:** 2016-12-10

**Authors:** Petr Badura, Dagmar Sigmundova, Erik Sigmund, Andrea Madarasova Geckova, Jitse P. van Dijk, Sijmen A. Reijneveld

**Affiliations:** 1 Faculty of Physical Culture, Institute of Active Lifestyle, Palacky University, Tr. Miru 117, 771 11 Olomouc, Czech Republic; 2 Department of Community and Occupational Medicine, University Medical Center Groningen, University of Groningen, Groningen, The Netherlands; 3 Olomouc University for Society and Health Institute, Palacky University, Olomouc, Czech Republic; 4 Department of Health Psychology, Faculty of Medicine, Safarik University, Kosice, Slovakia; 5 Graduate School Kosice Institute for Society and Health, Safarik University, Kosice, Slovakia

**Keywords:** Adolescents, Extracurricular activities, Substance use, Violence, Bullying, Truancy

## Abstract

**Objectives:**

The study aimed to assess the associations between participation in organized leisure-time activities (OLTA) and risk behaviors, and whether the associations differed by gender, age, and pattern of OLTA involvement.

**Methods:**

Data from the 2013/2014 Health Behaviour in School-Aged Children study on 10,279 11-, 13-, and 15-year-old Czech adolescents (49.2% boys) were used. We assessed the associations between OLTA participation and risk behaviors, and modification by age and gender.

**Results:**

OLTA participants were less likely to smoke, get drunk repeatedly, or skip school and, in contrast, more likely to get injured and fight repeatedly. The associations with lower occurrence of risk behaviors were the strongest for artists, while none was significant for adolescents participating only in team sports. Girls participating in OLTA had lower odds to smoke, get drunk, or skip school than boys, and these boys had higher odds to get injured or fight.

**Conclusions:**

OLTA participation is associated with lower occurrence of repeated substance use and truancy and inversely with higher odds for physical fights and injuries. Girls, in general, are at lower risk when participating in OLTA than boys.

## Introduction

Historically, adolescence has been perceived as a turbulent period typified by an inclination towards problematic behaviors, with the fundamental goal of overcoming this time period with least harm possible inflicted on an individual (Hall 1904). Lerner (2005) provided a detailed overview of the huge paradigm shift that has taken place since then. Today, young people are no longer considered only as ‘problems to be managed’ (Roth and Brooks-Gunn 2003). Contemporary science has instead adopted the perspective of developmental assets—youth individual strengths that need to be fostered through contextual resources (Bowers et al. 2014; Roth and Brooks-Gunn 2003). However, this approach does not omit the necessity of avoiding risk behaviors, and it has been hypothesized that such types of behaviors are negatively linked to healthy adolescent development (Lerner 2005).

There is solid evidence on the detrimental effects on health and well-being of various forms of risk behaviors, ranging from smoking tobacco (World Health Organization 2012) and consuming alcohol (World Health Organization 2014) to injuries as a consequence of risk-taking (Pickett et al. 2006) or acts of violence (Walsh et al. 2013). These health-compromising behaviors are often established in adolescence and persist into adulthood (Cook et al. 2015).

However, involvement in these behaviors is to a certain extent understood as typical for young people and may even have some positive consequences for youth development, such as learning experiences, integration into a clique, and stabilization of the social position acquired (Brady et al. 2008; Hurrelmann and Richter 2006). We should thus distinguish experimenting from serious risk-taking (Moffitt 1993). This was somewhat confirmed by Lewin-Bizan et al. (2010), who found that many adolescents labeled as developing healthily were also engaged in substance use or delinquency.

Although they account for only a small portion of time (Mahoney and Vest 2012), organized leisure-time activities (OLTA) seem to protect against risk behaviors (Farb and Matjasko 2012). OLTA participants are, in general, less likely to be involved in substance abuse, delinquency, or bullying others (Farb and Matjasko 2012; Riese et al. 2015). Time spent in OLTA might channel some stress-reduction efforts (Darling 2005), and affiliation to a certain club might negate the need for stabilization of one’s social position through risk behaviors (Viau et al. 2015).

Nonetheless, mere participation in OLTA obviously is not the only factor contributing to its association with a lower occurrence of risk behaviors. The type of OLTA seems to be important, too, with associations varying by type of OLTA. For instance, members of sport clubs, especially in regard to team sports, were observed to be more prone to drink alcohol (Linver et al. 2009) and act violently (Kreager 2007). This supports the notion that different types of activities provide adolescents with different developmental experiences, such as distinct opportunities for identity-related exploration or reliance in teamwork. This is attributable to fundamental nature (e.g., normative systems, goals, tasks, or performance conditions) of particular OLTA setting (Hansen et al. 2010). In addition, Takakura (2015) suggested that some types of OLTA (e.g., youth associations) could lead to a so-called dark side of social capital and consequently rather to adverse health-related outcomes, while from others, the youth may benefit. Moreover, the associations between OLTA involvement and risk behaviors tend to differ by gender, as the majority of the previous research reports boys benefiting more from such involvement (Fredricks and Eccles 2006; Metzger et al. 2011). Finally, OLTA have been shown to by vary socioeconomic status (Linver et al. 2009) and health-related behaviors as well (Currie et al. 2014).

Research on this topic has thus far been conducted mostly in the USA; therefore, we tried to verify if the associations would also be observed in a European context. This study aimed to assess the associations of participation in OLTA with repeated substance use, violent behaviors, truancy, and injuries among Czech adolescents aged 11, 13, and 15 years. Moreover, we examined whether the associations differ by gender, age, and pattern of OLTA involvement.

## Methods

### Sample and procedure

We used data from the Health Behaviour in School-Aged Children (HBSC) study conducted between April and June 2014 in the Czech Republic. Schools were the primary sampling unit. They were selected randomly from the database of the Czech Ministry of Education, Youth and Sports, after being stratified by region and type of school (primary vs. secondary schools). Out of 244 contacted schools, 243 granted consent (response rate 99.6%) to carry out the survey. One class per 5th, 7th, and 9th grade, which, in general, correspond to the age categories of 11, 13, and 15 years in the Czech Republic, was then selected at random at each of the participating schools. Questionnaires were administered during regular class time by trained research assistants in the absence of a teacher. Participation in the study was voluntary and anonymous, with no incentives offered to the participants. Parents or legal guardians of the adolescents were notified of the study and its purpose by the school management in advance and could withdraw their child if they disagreed. Prior to administration of the questionnaires, respondents were also given the opportunity to opt out of the study or skip questions that made them uncomfortable. The study design was approved by the Ethics Committee of the Faculty of Physical Culture, Palacky University, Olomouc (No. 17/2013).

We obtained data from 14,539 respondents (response rate 89.2%). Approximately, 10% of the children were not present at school during the survey. Thirty children refused to fill in the questionnaire (0.2%). First, in line with the HBSC protocol, we selected only adolescents aged 11, 13, and 15 years (*n* = 10,795). Next, we excluded 516 cases due to missing data on all OLTA items (*n* = 287), more than half (i.e. 5 or more) of risk behaviors (*n* = 4), contradictory responses on reported prevalence of risk behaviors in lifetime versus the last 30 days (*n* = 170), or other unlikely responses throughout the questionnaire (*n* = 55). For instance, a respondent who indicated drinking alcohol (get drunk or smoke cigarettes) more than three times in the past month but never during his or her lifetime was excluded from the analyses. The final sample comprised 10,279 adolescents.

### Measures

Participation in OLTA was assessed using the question: *In your free time, do you do any of these organized activities?* which was proven to have a good reliability (Bosakova et al. 2016). We investigated participation in the following six activities: *team sports*, *individual sports*, *art school*, *youth organizations*, *recreation/leisure centers or after*-*school clubs,* and *church meeting/singing*. Missing answers were considered to represent a ‘no’ unless all six OLTA items were missing. Then, the respondent was excluded (*n* = 287). First, the OLTA participation was investigated using a dichotomous variable (participation in *at least one OLTA* vs. in *no OLTA*). Next, the respondents were split into five groups of OLTA participation patterns (Table 1) based on a cluster analysis (Badura et al. 2015).

**Table 1:** Description of the respondents’ patterns (clusters) of the organized leisure-time activity participation; 2013/2014 Health Behaviour in School-Aged Children study in the Czech Republic

	Patterns of OLTA derived by the cluster analysis				
	All rounders (*n* = 3183)	Artists (*n* = 1921)	Ind. sports (*n* = 1344)	Team sports (*n* = 1896)	Inactive (*n* = 1935)
Gender					
Boys	1539 (48.4%)	495 (25.8%)	720 (53.6%)	1389 (73.3%)	913 (47.2%)
Girls	1644 (51.6%)	1426 (74.2%)	624 (46.4%)	507 (26.7%)	1022 (52.8%)
Age					
11 years old	1280 (40.2%)	689 (35.9%)	347 (25.8%)	507 (26.7%)	419 (21.7%)
13 years old	1108 (34.8%)	664 (34.6%)	462 (34.4%)	657 (34.7%)	569 (29.4%)
15 years old	795 (25.0%)	568 (29.6%)	535 (39.8%)	732 (38.6%)	947 (48.9%)
FAS					
Low	849 (27.1%)	514 (27.1%)	334 (25.2%)	496 (26.5%)	533 (27.9%)
Medium	1002 (32.0%)	578 (30.4%)	469 (35.3%)	627 (33.5%)	582 (30.5%)
High	1285 (41.0%)	811 (42.6%)	525 (39.5%)	747 (39.9%)	769 (41.7%)
Types of activities					
Team sports	1584 (49.8%)	748 (38.9%)	685 (51.0%)	1896 (100%)	0
Individual sports	1134 (35.6%)	618 (32.2%)	1344 (100%)	0	0
Arts	1434 (45.1%)	1921 (100%)	0	0	0
Youth organizations	1342 (42.2%)	0	0	0	0
Leisure centers^a^	1962 (61.6%)	0	0	0	0
Church meetings	781 (24.5%)	0	0	0	0
Number of activities done concurrently					
1 activity	493 (15.5%)	836 (43.5%)	659 (49.0%)	1896 (100%)	0
2 activities	1153 (36.2%)	804 (41.9%)	685 (51.0%)	0	0
3 activities	957 (30.1%)	281 (14.6%)	0	0	0
4 or more activities	580 (18.2%)	0	0	0	0

% represents percentage in particular OLTA cluster, *FAS* Family Affluence Scale, *OLTA* organized leisure-time activities; there were no missing values for OLTA in the final data set

^a^This type of OLTA includes recreation or leisure centers and after-school clubs

As indicators of risk behaviors, we selected eight distinct phenomena with the following cut-off points: *current smoking* (at least once a week), *alcohol consumption* (at least 3 days in the last 30 days), *drunkenness* (at least twice in the last 30 days), *injuries* (at least twice in the past 12 months), *physical fighting* (at least twice in the past 12 months), *truancy* (at least once in the past 12 months), and *bullying others*—*perpetration* and *being bullied*—*victimization* (at least twice a month). Using the last two items, as Solberg et al. (2007) recommended to distinguish between bullies and bully-victims (i.e., those being both bully perpetrators and victims), we derived a categorical variable of youth involved in bullying others (*bully* and *bully-victim* vs. *those not involved in bullying others*). Cut-off points regarding substance use and fighting were set to capture only more severe (recurrent, i.e., more than once) forms and to avoid undesired inclusion of their occasional occurrence. Items were part of the HBSC mandatory questionnaire, except for truancy. This was assessed as ‘*During the past 12* *months, did you skip school without a proper excuse for at least a whole day?*’ The detailed description and scientific rationale of the other items can be found in the HBSC International Protocol (Currie et al. 2014).

Socioeconomic status of adolescents’ families, as a confounder, was measured by the Family Affluence Scale (FAS) developed for the purposes of the HBSC study (Currie et al. 2014). The responses on six items exploring various indicators of family socioeconomic status (car ownership, holidays abroad, having one’s own bedroom, number of computers in the household, number of bathrooms, and dishwasher ownership) were summed up and transformed to a fractional rank score ranging from 0 to 1. The score was then trichotomized to classify respondents into groups of low (0–0.333), medium, (0.334–0.666), and high (0.667–1) socioeconomic status.

### Statistical analyses

First, we described the composition of the sample and its involvement in various risk behaviors. Second, using binary logistic regressions, we assessed the associations of the dichotomized overall OLTA variable (at least one activity vs. none) with cigarette smoking, alcohol use, drunkenness, involvement in physical fights, truancy, and injuries. The association of OLTA participation with bullying perpetration was analyzed using multinomial logistic regression. In the first step, we assessed crude associations per risk behavior and bullying categories, respectively (Model 1). Next, we adjusted for age and gender (Model 2), and then also for FAS (Model 3). At last, we tested interaction effects of gender (Model 4) and age (Model 5) on these associations. Then, using a two-step cluster analysis, we derived five distinct clusters of adolescents based on their pattern of OLTA participation. This number of clusters was the smallest possible that led to a reasonably high homogeneity within the clusters and reasonably high differences between particular clusters, shown by an average silhouette width over the value of 0.5 (Badura et al. 2015). All the regression analyses were then run again for each separate cluster of OLTA as an independent variable. The data were analyzed using ordinary single-level regression, because multilevel analyses yielded no indication of clustering by school. Prior to conducting the regression analyses, we assessed the random variance of specific risk behaviors at the level of schools, and for none of them, it was statistically significant. The statistical analyses were carried out using IBM SPSS 22 for Windows (IBM Corp. Released 2013) and MLwiN Version 2.02 (Centre for Multilevel Modelling, University of Bristol).

## Results

As is apparent from Table 2, relatively few adolescents engaged in recurrent substance use and were involved bullying—either as a bully or bully-victim. The overall prevalence was the highest for fighting, with nearly one in four getting into a fight at least twice in the last year.

**Table 2:** Description of the study population: rate of respondents’ involvement in risk behaviors and number of missing values per variable; 2013/2014 Health Behaviour in School-Aged Children study in the Czech Republic

	*n*	%	Missing values
Gender			
Boys	5056	49.2	0
Girls	5223	50.8	
Age			
11 years old	3242	31.5	0
13 years old	3460	33.7	
15 years old	3577	34.8	
FAS			
Low	2726	26.9	131
Medium	3258	32.1	
High	4164	41.0	
Risk behaviors			
Smoking ≥1 ×/weekly	681	6.6	57
Alcohol ≥3 ×/last 30 days	926	9.0	241
Drunkenness ≥2 ×/last 30 days	257	2.5	245
Injuries ≥2 ×/last year	1764	17.2	23
Fighting ≥2 ×/last year	2273	22.1	46
Truancy ≥1 ×/last year	1507	14.7	42
Bully ≥2 ×/monthly	225	2.2	151
Bully-victim ≥2 ×/monthly	90	0.9	151

% valid percent in the total sample (*n* = 10,279), *FAS* Family Affluence Scale

Table 3 presents the odds ratios (OR) and 95% confidence intervals (CI) for the associations of the dichotomized OLTA participation variables with the risk behaviors. Adolescents participating in at least one OLTA were less likely to smoke, get drunk, or skip school than their inactive peers, both crude (Model 1) and also after adjustment for differences in gender and age (Model 2), as well as FAS (Model 3). On the other hand, we observed higher odds for fighting and getting injured among those involved in OLTA. No association was found with either of the bullying categories (not shown).

**Table 3:** Association of dichotomized participation variables with risk behaviors: odds ratios (OR) and 95% confidence intervals (CI) for active vs. inactive adolescents (reference category, with OR = 1), 2013/2014 Health Behaviour in School-Aged Children study in the Czech Republic

	Model 1 (univariable) ≥1 activity vs. inactive		Model 2 (adjusted for gender and age) ≥1 activity vs. inactive		Model 3 (adjusted for gender, age and FAS)		Model 4 (interaction of OLTA participation with gender, adjusted for age, and FAS)						
					≥1 activity vs. inactive		Main effect of OLTA (≥1 activity vs. inactive)		Main effect of gender (B vs. G)		Interaction of OLTA and gender (OLTA participants B vs. G)		
	OR	95% CI	OR	95% CI	OR	95% CI	OR	95% CI	OR	95% CI	OR	95% CI	*P* value
Smoking (≥1×/weekly)	**0.51**	**0.43–0.60**	**0.70**	**0.59–0.84**	**0.70**	**0.59–0.84**	**0.54**	**0.43–0.68**	**0.52**	**0.38–0.71**	**1.91**	**1.32–2.76**	**<.001**
Alcohol (≥3×/last 30 days)	**0.73**	**0.62–0.86**	0.94	0.80–1.11	0.94	0.80–1.11	**0.77**	**0.61–0.97**	1.14	0.86–1.53	**1.48**	**1.06–2.07**	**0.021**
Drunkenness (≥2×/last 30 days)	**0.50**	**0.38–0.65**	**0.68**	**0.51–0.89**	**0.68**	**0.52–0.89**							
Injuries (≥2×/last year)	**1.78**	**1.53–2.07**	**1.87**	**1.60–2.18**	**1.87**	**1.60–2.18**	**1.54**	**1.25–1.89**	0.80	0.60**–**1.06	**1.51**	**1.11–2.06**	**0.009**
Fighting (≥2×/last year)	**1.27**	**1.12–1.45**	**1.17**	**1.03–1.34**	**1.18**	**1.03–1.34**	0.95	0.76–1.19	**3.27**	**2.55–4.20**	**1.37**	**1.04–1.81**	**0.026**
Truancy (≥1×/last year)	**0.85**	**0.75–0.98**	**0.84**	**0.73–0.96**	**0.84**	**0.73–0.96**	**0.72**	**0.60–0.87**	0.85	0.67–1.09	**1.37**	**1.04–1.79**	**0.028**

*P* values based on logistic regression analyses

*B* boys, *G* girls, *OR* odds ratio, *CI* confidence interval, *OLTA* organized leisure-time activities, *FAS* Family Affluence Scale; ORs and 95% CIs for Model 4 are presented only when the interaction effect of gender with OLTA participation was statistically significant

Statistically significant (*p* < 0.05) odds ratios and 95% confidence intervals are indicated in bold

**Fig. 1:**
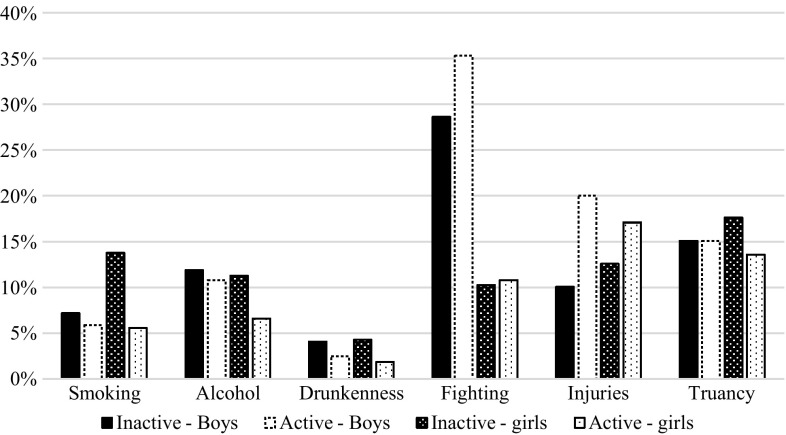
Percentages of adolescents active in organized leisure-time activities and those inactive who reported being involved in specific risk behaviors by gender (total sample, *n* = 10,279); 2013/2014 Health Behaviour in School-Aged Children study in the Czech Republic

The interaction effects with gender on the association of participation in OLTA with risk behaviors were tested next. Apart from drunkenness, all of them were significant. The associations between OLTA participation and less frequent smoking, alcohol consumption, and truancy were stronger in girls (Model 4, see also Fig. 1), showing that girls participating in at least one OLTA are less likely to be engaged in a risk behavior, whereas this participation does not make a difference in boys. On the other hand, boys participating in at least one OLTA are more likely to get injured or involved in physical fights than girls participating in OLTA compared with their nonparticipating counterparts. No OLTA–gender interactions were found concerning the bullying categories. We did not observe any interactions with age either (Model 5 not shown). The only exception was that 13-year-old OLTA participants were significantly less likely to be bully than those aged 15 (OR = 0.35, 95% CI = 0.17–0.73).

Table 4 presents the results of the logistic regressions using clusters of OLTA as independent variables, with the inactive cluster as the reference category. After adjustment for age, gender, and FAS (Model 3), the all-rounders, artists, and individual sports clusters, but not team sports, had lower odds for regular smoking and repeated drunkenness in the last 30 days. No association was observed between OLTA clusters and recent alcohol consumption, except in the crude model. In all clusters, active adolescents were significantly more likely to get injured. The all-rounders and team sports cluster members had also higher odds of getting involved in physical fights more than once in the last year. Regarding bullying, we found no significant association with being a bully (perpetrator) after adjusting for gender, age, and FAS (Model 3). However, artists had significantly lower odds of being a bully-victim.

**Table 4:** Association of participation in organized leisure-time activities (clusters of activity pattern) with risk behaviors: odds ratios and 95% confidence intervals for active vs. inactive adolescents (inactive cluster is the reference category); 2013/2014 Health Behaviour in School-Aged Children study in the Czech Republic

	Smoking ≥1 ×/weekly	Alcohol ≥3 ×/last 30 days	Drunkenness ≥2 ×/last 30 days	Injuries ≥2 ×/last year	Fighting ≥2 ×/last year	Truancy ≥1 ×/last year	Bully ≥2 ×/monthly	Bully-victim ≥2 ×/monthly
Model 1 (univariable)								
Inactive	1 (ref)	1 (ref)	1 (ref)	1 (ref)	1 (ref)	1 (ref)	1 (ref)	1 (ref)
All rounders	0.36*** (0.28–0.45)	0.62*** (0.51–0.75)	0.40*** (0.28–0.57)	1.69*** (1.43–2.00)	1.43*** (1.24–1.65)	0.89 (0.76**–**1.04)	0.90 (0.61**–**1.31)	1.03 (0.60**–**1.75)
Artists	0.43*** (0.33–0.55)	0.61*** (0.49–0.76)	0.33*** (0.21–0.51)	1.52*** (1.26–1.83)	0.75** (0.64–0.90)	0.67*** (0.56–0.81)	0.62* (0.38–0.99)	0.22** (0.09–0.59)
Individual sports	0.60*** (0.46–0.77)	0.83 (0.66**–**1.04)	0.42*** (0.27–0.67)	2.00*** (1.64–2.42)	1.21* (1.02–1.45)	0.88 (0.73**–**1.07)	1.15 (0.74**–**1.80)	0.46 (0.20**–**1.08)
Team sports	0.78* (0.63–0.97)	1.00 (0.82**–**1.22)	0.91 (0.66**–**1.26)	2.04*** (1.71–2.45)	1.67*** (1.43–1.95)	0.97 (0.81**–**1.15)	1.14 (0.76**–**1.71)	0.88 (0.48**–**1.64)
Model 2 (adjusted for gender and age)								
Inactive	1 (ref)	1 (ref)	1 (ref)	1 (ref)	1 (ref)	1 (ref)	1 (ref)	1 (ref)
All rounders	0.58*** (0.45–0.73)	0.90 (0.74**–**1.11)	0.64* (0.45–0.92)	1.82*** (1.53–2.16)	1.32*** (1.14–1.53)	0.87 (0.74**–**1.01)	1.03 (0.70**–**1.52)	0.91 (0.53**–**1.56)
Artists	0.58*** (0.44–0.75)	0.89 (0.71**–**1.12)	0.47*** (0.30–0.74)	1.63*** (1.35–1.98)	0.96 (0.80**–**1.15)	0.66*** (0.55–0.80)	0.80 (0.49**–**1.29)	0.23** (0.09–0.61)
Individual sports	0.70** (0.53–0.91)	0.91 (0.72**–**1.15)	0.48** (0.30–0.77)	2.03*** (1.67–2.47)	1.10 (0.91**–**1.31)	0.88 (0.72**–**1.07)	1.15 (0.74**–**1.80)	0.43 (0.18**–**1.01)
Team sports	1.00 (0.80**–**1.26)	1.05 (0.85**–**1.29)	1.06 (0.76**–**1.49)	2.05*** (1.71–2.46)	1.19* (1.01–1.40)	0.96 (0.80**–**1.14)	1.01 (0.68**–**1.55)	0.75 (0.40**–**1.40)
Model 3 (adjusted for gender, age, and FAS)								
Inactive	1 (ref)	1 (ref)	1 (ref)	1 (ref)	1 (ref)	1 (ref)	1 (ref)	1 (ref)
All rounders	0.58*** (0.46–0.73)	0.91 (0.74**–**1.11)	0.64* (0.45–0.91)	1.82*** (1.53–2.16)	1.32*** (1.14–1.54)	0.87 (0.74**–**1.02)	1.05 (0.71**–**1.56)	0.91 (0.53**–**1.57)
Artists	0.58*** (0.44–0.75)	0.89 (0.70**–**1.12)	0.47*** (0.30–0.74)	1.63*** (1.35–1.97)	0.96 (0.80**–**1.15)	0.66*** (0.55–0.80)	0.81 (0.50**–**1.31)	0.23** (0.09–0.61)
Individual sports	0.70** (0.54–0.92)	0.91 (0.72**–**1.16)	0.48** (0.30–0.77)	2.03*** (1.67–2.47)	1.09 (0.91**–**1.31)	0.88 (0.73**–**1.07)	1.15 (0.73**–**1.81)	0.43 (0.18**–**1.02)
Team sports	1.01 (0.80**–**1.27)	1.05 (0.85**–**1.30)	1.07 (0.76**–**1.50)	2.05*** (1.71–2.46)	1.19* (1.01–1.40)	0.96 (0.80**–**1.14)	1.05 (0.69**–**1.59)	0.75 (0.40**–**1.41)

*FAS* Family Affluence Scale

Statistically significant (*p* < 0.05) odds ratios and 95% confidence intervals are indicated in bold

* *P* < 0.05, ** *P* < 0.01, *** *P* < 0.001—*P* values based on the logistic and multinomial regression analyses

**Fig. 2:**
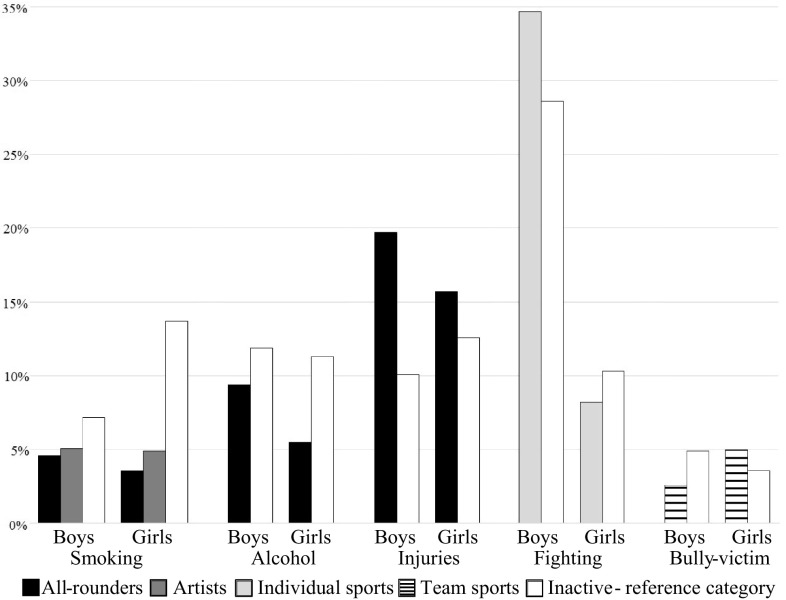
Percentages of adolescents per organized leisure-time activity cluster who reported being involved in specific risk behaviors, in cases where the interactions of the cluster with gender were statistically significant (total sample, *n* = 10,279); 2013/2014 Health Behaviour in School-Aged Children study in the Czech Republic

Only a few of the interactions of gender with the associations between OLTA clusters and risk behaviors were statistically significant. Membership of the all-rounder (OR = 2.55, 95% CI = 1.58–4.13) and artists (OR = 2.00, 95% CI = 1.12–3.57) clusters was associated with significantly higher odds for smoking in boys than in girls. Although the interaction effects with gender regarding the remaining risk behaviors were not statistically significant in terms of the overall variable *p* value for all OLTA categories combined, we did observe interactions of gender after splitting the OLTA participants into separate clusters. Compared with the inactive cluster, boys in the all-rounders cluster had higher odds of drinking alcohol than all-rounder girls (OR = 1.65, 95% CI = 1.10–2.48) and, likewise, of fighting when in the individual sports cluster (OR = 1.92, 95% CI = 1.26–2.92). Affiliation to the all-rounder cluster was furthermore associated with higher risk of injuries, especially in boys (OR = 1.67, 95% CI = 1.19–2.35). Selected gender-stratified percentages of adolescents involved in risk behaviors per OLTA cluster are shown in Fig. 2. The interactions of OLTA clusters with age categories were not statistically significant (Model 5, not shown).

## Discussion

Participation in OLTA was associated with lower odds for regular smoking, recurrent drunkenness, and truancy. In contrast, OLTA participants were more likely to get injured and to fight repeatedly. Associations for reduced alcohol consumption were less consistent and non-significant after adjustment for age, gender, and FAS. However, the associations slightly varied by specific OLTA clusters, with artists (involved only in art activities or combining arts and sports) being the least prone to individual risk behaviors and at less risk of getting injured. In contrast, membership to the team sports cluster (those involved only in team sports) was not significantly associated with reduced risk occurrence. These findings, in general, are in accord with the previous research conducted mostly in the USA. However, we found a relatively consistent effect of gender on the associations between OLTA and risk behaviors. Unlike the previous US studies (Fredricks and Eccles 2006; Metzger et al. 2011), we rather observed girls profiting from OLTA participation in terms of being exposed to a lower chance of getting involved in one of the examined risk behaviors.

We found that adolescents involved in OLTA were, in general, less likely to be engaged in risk behaviors than their uninvolved peers. This finding is in agreement with the previous studies linking OLTA to less health-compromising behaviors (Bohnert and Garber 2007; Farb and Matjasko 2012). This could be explained by the fact that adolescents tend to engage in risk behaviors less often, because they spend a considerable amount of time under influential adult supervision (Bohnert and Garber 2007). Moreover, the positive identity-related experiences and goal-directed behaviors as a consequence of OLTA participation are thought to protect against risk behaviors (Palen and Coatsworth 2007). It seems that the structured content of OLTA and guidance of adult leaders make adolescents less prone to get involved in risk behaviors.

We also observed lower odds for truancy in adolescents participating in OLTA, which complements the finding that youth with mostly unstructured leisure time reach the highest levels of truancy (Nelson and Gastic 2009). There is evidence of higher attachment to school and better general school performance among OLTA participants (Fredricks 2012). They are also able to maintain healthier relationships both with their peers and non-familial adults (Crean 2012; Schaefer et al. 2011). As a consequence, they, perhaps, try to avoid school duties less often (Palen and Coatsworth 2007) and feel better in the social context of school, which could, in turn, lead to the lower rate of active adolescents skipping school.

On the other hand, the odds of getting injured and being involved in physical fights were higher in those participating in OLTA, particularly in boys. All the active clusters contained adolescents participating in sports, and this finding would fit quite well the picture of sports as an environment in which various conflicts frequently arise (Martin et al. 2014). Although in sports, adolescents also learn to cope with conflicts, they are still focused on efforts of physical domination over the opponent and present opportunities to ‘compare muscles’. This, in combination with eventual conflicts, might partly underlie our finding on higher odds of fighting when involved in OLTA. The higher rate of injuries seems to be logical as well, as sports markedly increase the risk of medically attended injuries (Maffulli et al. 2011).

Our consistent finding that girls are less engaged in risk behaviors when participating in OLTA is, to the best of our knowledge, unprecedented. The previous research found OLTA participation associated either with reduced risk behaviors in boys (Fredricks and Eccles 2006; Metzger et al. 2011) or, oppositely, increased risk in girls (Linville and Huebner 2005). It has been shown that girls in adolescence believe more in the legitimacy of an adult authority (Kuhn and Laird 2011). This may decrease their involvement in risk behaviors when supervised by an adult, such as a coach or leader.

As in a recent study (Takakura 2015), we observed differences by OLTA participation patterns. The artist cluster yielded the strongest associations with less substance use and none with fighting. The team sports cluster members, in contrast, showed no significant association with reduced risk behaviors and were only more likely to get injured or fight. Some young athletes, especially those involved in team sports, were, indeed, shown to be more prone to aggressive conduct (Kreager 2007) or higher rates of alcohol consumption (Farb and Matjasko 2012). In contrast, Denault et al. (2009) observed the intensity of participation in art activities to be predictive of lower alcohol consumption, and Sharp et al. (2015) found that children taking part in multiple activities reported significantly lower levels of substance use. This is in line with our findings and only underpins the assumption that the actual type (pattern in our case) of activity matters (Badura et al. 2015).

### Strengths and limitations

The large and representative sample is the most important strength of this study. Furthermore, it was based on the well-established and recognized HBSC study, with a strong methodological background regarding data collection procedures and construction of the questionnaire, which is being developed by an international expert team on a continuous basis.

This study also has some limitations. First, due to its cross-sectional design, it is impossible to determine causality of the associations. Second, the use of self-reported data is, in general, more susceptible to being biased. However, the HBSC mandatory items on substance use, injuries, and violent behaviors have been shown to have validity and reliability (Currie et al. 2014). Third, we lacked more detailed information on specific types of OLTA, as it has been proven that, e.g., there is a difference between American football and baseball in aggressive behaviors (Kreager 2007), while our measures did not allow capturing such differences. Finally, we also did not have data on other dimensions of OLTA, such as intensity, duration, and engagement, which might play unique roles and could have affected the associations found.

### Implications

In general, the findings of this study demonstrate the associations between OLTA participation and lower occurrence of regular substance use and truancy. This indicates that conclusions drawn previously in the US context might be applicable to the European context as well. The structured content of OLTA could, therefore, serve as one of the options to address these issues, especially in girls, who seem to profit more from their participation in terms of lower occurrence of risk behaviors. In particular, art activities, either alone or in combination with sports, appear to be the most beneficial OLTA context. Future research should concentrate on revealing the causal pathways between OLTA participation and risk behaviors.

### Conclusions

OLTA participants are less likely to smoke regularly, get drunk repeatedly, and skip school. On the other hand, OLTA involvement is associated with higher risk of physical fighting and injuries. Girls, in general, seem to be at lower risk of risk behaviors when participating in OLTA than boys. However, the specific OLTA pattern is important, because we found no significant association with reduced occurrence of risk behaviors among adolescents involved only in team sports.
